# Genome-Wide Characterization of NBS-Encoding Genes in Watermelon and Their Potential Association with Gummy Stem Blight Resistance

**DOI:** 10.3390/ijms20040902

**Published:** 2019-02-19

**Authors:** Md Zahid Hassan, Md Abdur Rahim, Hee-Jeong Jung, Jong-In Park, Hoy-Taek Kim, Ill-Sup Nou

**Affiliations:** Department of Horticulture, Sunchon National University, Suncheon 57922, Korea; zhassan.pstu@gmail.com (M.Z.H.); rahimgepb@sau.edu.bd (M.A.R.); gml79wjd@scnu.ac.kr (H.-J.J.); jipark@sunchon.ac.kr (J.-I.P.); htkim@sunchon.ac.kr (H.-T.K.)

**Keywords:** gummy stem blight, watermelon, resistance, NBS genes, expression

## Abstract

Watermelon (*Citrullus lanatus*) is a nutritionally rich and economically important horticultural crop of the Cucurbitaceae family. Gummy stem blight (GSB) is a major disease of watermelon, which is caused by the fungus *Didymella bryoniae*, and results in substantial economic losses in terms of yield and quality. However, only a few molecular studies have focused on GSB resistance in watermelon. Nucleotide binding site (NBS)-encoding resistance (*R*) genes play important roles in plant defense responses to several pathogens, but little is known about the role of NBS-encoding genes in disease resistance in watermelon. The analyzed NBS-encoding *R* genes comprises several domains, including Toll/interleukin-1 receptor(TIR), NBS, leucine-rich repeat (LRR), resistance to powdery mildew8(RPW8) and coiled coil (CC), which are known to be involved in disease resistance. We determined the expression patterns of these *R* genes in resistant and susceptible watermelon lines at different time points after *D. bryoniae* infection by quantitative RT-PCR. The *R* genes exhibited various expression patterns in the resistant watermelon compared to the susceptible watermelon. Only six *R* genes exhibited consistent expression patterns (*Cla001821*, *Cla019863*, *Cla020705*, *Cla012430*, *Cla012433* and *Cla012439*), which were higher in the resistant line compared to the susceptible line. Our study provides fundamental insights into the NBS-LRR gene family in watermelon in response to *D. bryoniae* infection. Further functional studies of these six candidate resistance genes should help to advance breeding programs aimed at improving disease resistance in watermelons.

## 1. Introduction

Watermelon (*Citrullus lanatus*) is one of the most important cucurbit crops worldwide. This crop is well known in all tropical and subtropical regions of the world, where it is primarily grown for the fresh consumption of the juicy, sweet flesh of its mature fruit [1]. Watermelon plays a significant role in human health by providing important nutritional compounds, such as sugars and lycopene, and cardiovascular health-promoting amino acids, including arginine, glutathione and citrulline [2]. 

However, watermelon is frequently affected by several pathogens and insects, including fungi, bacteria, viruses and aphids. Gummy stem blight (GSB) is one of the most destructive diseases of watermelons. GSB is caused by the soil, seed and airborne fungus *Didymella bryoniae* [3,4]. This disease also affects other cucurbit crops, including melon, cucumber and squash [5,6]. GSB in watermelon causes crown blight, stem cankers and extensive defoliation, with symptoms detected in cotyledons, hypocotyls, leaves and fruits. This results in severe economic losses in the field and fruit losses during storage [4,7]. It is difficult to properly control GSB using chemical methods and the frequent use of fungicides is not desirable due to their negative impact on the environment [4,8]. Consequently, developing GSB-resistant watermelon cultivars containing major resistance genes through gene pyramiding represents the most environmentally sustainable and economically viable means of GSB management [4,9]. 

Due to the agricultural importance of this disease, early studies have focused on identifying the sources of genetic resistance to GSB [10]. However, few such sources have been reported in watermelon [4,7]. The identification of linked markers is essential for exploring GSB resistance in watermelon genotypes from diverse germplasm. However, no molecular studies of GSB resistance in watermelon have thus far been reported and current efforts are still focused on developing a GSB-resistant watermelon cultivar. 

Resistance (*R*) genes play important roles in plant immune systems in response to various pathogens and insects, including viruses, bacteria, fungi, aphids and nematodes [11]. Disease resistance in plants involves the interaction between the *avirulence* (*avr*) genes of the pathogen and specific disease resistance (*R*) genes of plants, with such interactions described by the gene-for-gene model [12,13]. This form of plant resistance can be lost due to the development of new races of pathogens via evolution or as a consequence of the evolutionary loss of *R* genes [14].

Most *R* genes in plants encode proteins that are comprised of a nucleotide-binding site (NBS) and leucine-rich repeats (LRRs), which play vital roles in plant–pathogen recognition [15]. The NBS domains, which bind to and hydrolyze adenosine triphosphate (ATP) or guanosine triphosphate (GTP), are involved in signaling, whereas LRRs are highly adaptable structural domains that are responsible for protein–protein interactions [15]. Plant NBS-LRR proteins can be classified into two subgroups based on the identity of the sequences that precede the NBS domain: TIR-NBS-LRR (TNL) proteins that have Toll-like domains and CC-NBS-LRR (CNL) proteins that are characterized by their coiled-coil domains. Watermelon contains 44 NBS-LRR genes, as revealed by genomic analysis [1]. *R* genes have been identified in a number of plant species, including *Arabidopsis thaliana* [16], rice [17,18], melon [19], cucumber [20] and apple [21]. Initially, we identified GSB-resistant watermelon lines through an extensive bioassay. Since NBS-encoding *R* genes have been reported to confer resistance against various pathogens and insects in different plant species, the *R* genes were explored in watermelon. Further, the availability of the complete genome sequences of *C. lanatus* allowed us to systematically analyze the NBS-encoding *R* genes in watermelon.

However, little is known about NBS-encoding *R* genes in resistant and susceptible watermelon lines/cultivars. Therefore, in this study, we analyzed the expression patterns of NBS-encoding genes in *D. bryoniae*-resistant and -susceptible watermelon lines to identify candidate *R* genes conferring resistance to GSB, which could be highly useful for breeding programs. 

## 2. Results

### 2.1. Distribution of 44 NBS-Encoding Genes in Watermelon Chromosomes

**Figure 1:**
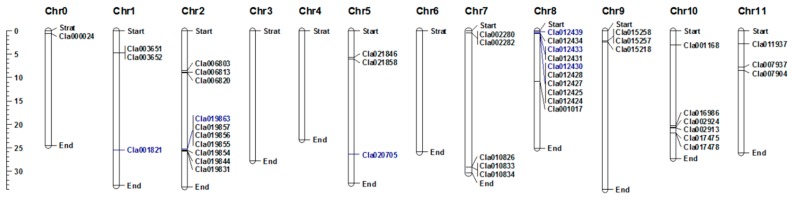
Distribution of the 44 NBS-encoding *R* genes on the watermelon chromosomes. The genes shown in blue are the candidate NBS-encoding genes for gummy stem blight resistance in watermelon. The positions of genes on the watermelon chromosomes were drawn using MapChart software [22].

A total of 44 NBS-encoding *R* genes were previously identified in the watermelon genome [1]. In this study, we found that the genes are differentially expressed in susceptible compared to resistant *C. lanatus* lines. Forty-four *R* genes are distributed across nine *C. lanatus* chromosomes, ranging from 1 to 10 genes per chromosome (Figure 1, Table 1). The highest number of NBS-encoding genes was found on Chr2 (*Cla006803*, *Cla006813*, *Cla006820*, *Cla019844*, *Cla019831*, *Cla019854*, *Cla019855*, *Cla019856*, *Cla019857* and *Cla019863*) and Chr8 (*Cla001017*, *Cla012424*, *Cla012425*, *Cla012427*, *Cla012428*, *Cla012430*, *Cla012431*, *Cla012434* and *Cla012439*), whereas the lowest number was found on Chr0 (*Cla000024*). Chr1, Chr5, Chr9 and Chr11 each contain three NBS-encoding genes, while Chr7 contains five and Chr10 contains six genes (Figure 1).

### 2.2. Exon–Intron Structure

**Figure 2:**
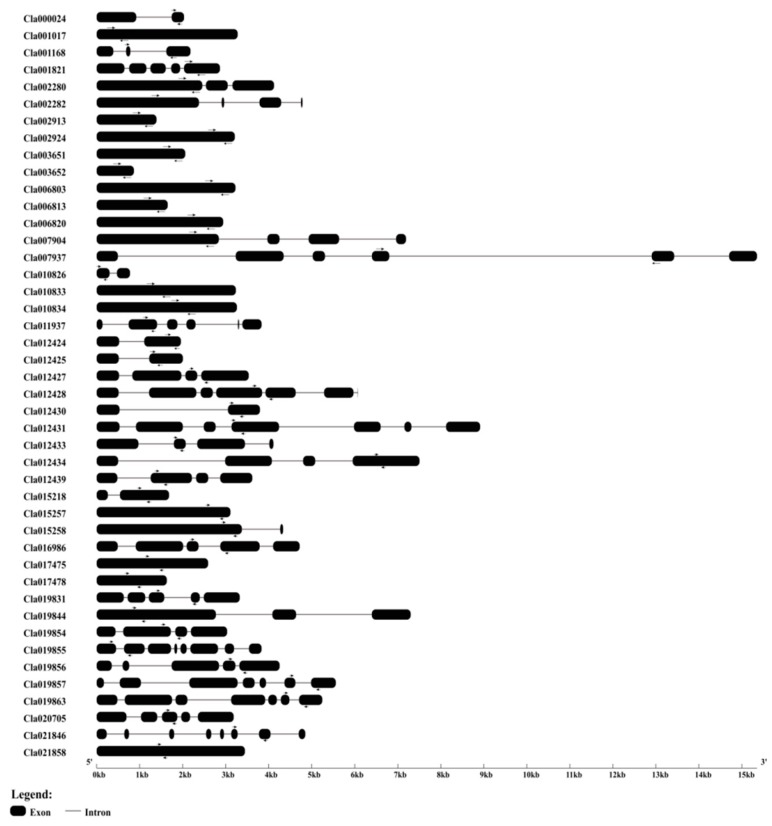
Exon–intron structures of the 44 NBS-encoding *R* genes from watermelon. Rectangles and gray lines indicate exons and introns, respectively. Left and right arrows indicate position of forward and reverse primers, respectively.

To better understand the genomic structures of the 44 NBS-encoding *R* genes, we generated exon–intron diagrams of the genes by comparing their coding sequences with the corresponding genomic sequences using the online tool GSDS2.0 (http://gsds.cbi.pku.edu.cn/). The number of exons per gene was 1–8 (Figure 2). The highest number of exons was found in *Cla019855* and *Cla021846*, while the lowest was found in *Cla001017*, *Cla006813*, *Cla012424*, *Cla003651*, *Cla003652*, *Cla006803*, *Cla002913*, *Cla006820*, *Cla010833*, *Cla010834*, *Cla015257*, *Cla017475*, *Cla017478* and *Cla021858*. Three genes (*Cla007937*, *Cla011937* and *Cla012428*) contain six exons while three other genes (*Cla012431*, *Cla019857* and *Cla019863*) contain seven exons. The number of introns per gene was 0–7. *Cla019855* and *Cla021846* contain the most introns, whereas 14 genes lack introns (Figure 2). 

### 2.3. Conserved Domain and Motif Analysis

**Figure 3:**
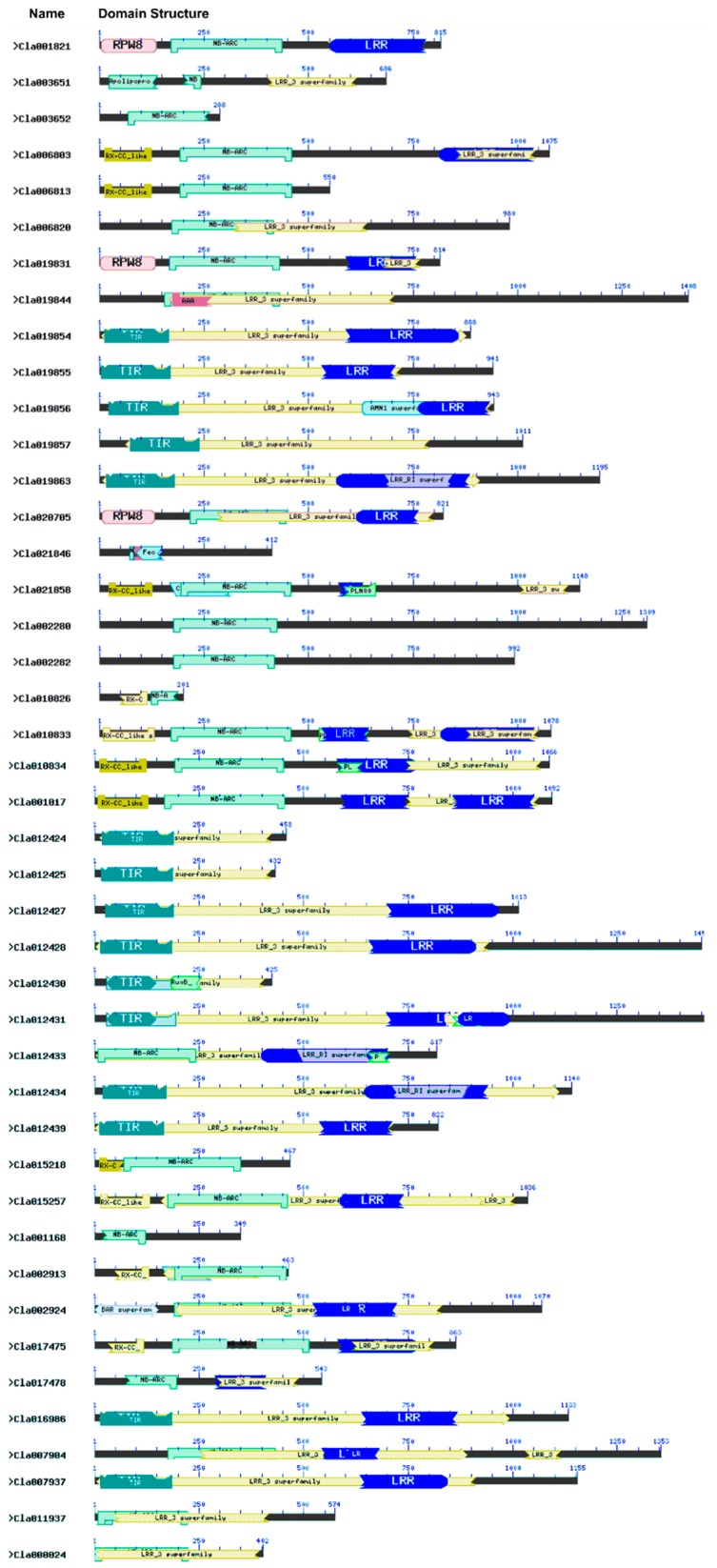
Domains present in NBS proteins in watermelon. The conserved domains were identified using the Conserved Domain Database (CDD) of NCBI against the Pfam v30.0 database (https://www.ncbi.nlm.nih.gov/Structure/cdd/wrpsb.cgi). Detailed descriptions of these domains are provided in Table 2. Specific domains in each protein are shown in the diagram.

**Figure 4:**
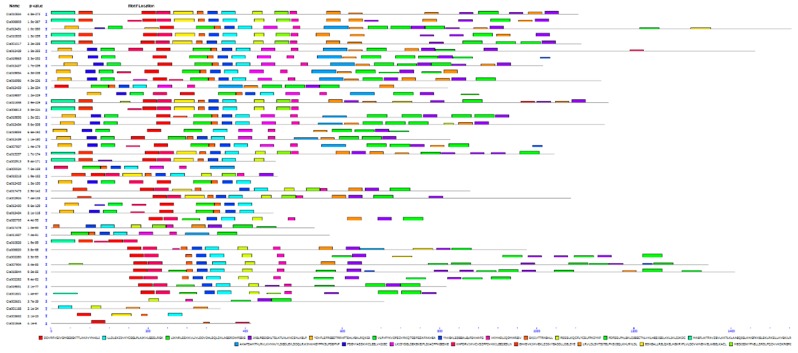
Conserved motifs of NBS-encoding *R* genes in the watermelon genome. Motifs are indicated by different colored rectangles. Detailed information is provided in Table 4.

We analyzed the conserved domains of the 44 NBS-encoding *R* genes using the Conserved Domain Database (CDD) of NCBI and the Pfam protein database v30.0 (https://www.ncbi.nlm.nih.gov/structure/cdd/wrpsb.cgi). These results are shown in Figure 3 and Table 2. All 44 proteins have a highly conserved NBS (NB-ARC) domain. The *R* genes were grouped in different classes based on the presence of the following conserved domains: (i) NBS, (ii) NBS-LRR, (iii) LRR, (iv) RPW8-NBS-LRR, (v) TIR, (vi) TIR-LRR, (vii) CC-NBS and (viii) CC-NBS-LRR (Table 3). Three genes (*Cla001821*, *Cla019831* and *Cla020705*) encode proteins with both RPW8 and NBS-LRR domains. We subjected the 44 NBS proteins to motif analysis using the MEME Suite (http://meme-suite.org/tools/meme). These results are shown in Figure 4 and Table 4. We detected 20 conserved motifs, each being comprised of over 13 amino acids. The greatest number of motifs was identified in the CC-NBS-LRR domain-containing gene *Cla021858*, whereas the fewest were detected in *Cla001168* and *Cla021846*. 

### 2.4. Synteny Analysis of 44 NBS-Encoding R Genes of C. lanatus Compared with Cucumis melo, Cucumis sativus, and A. thaliana

**Figure 5:**
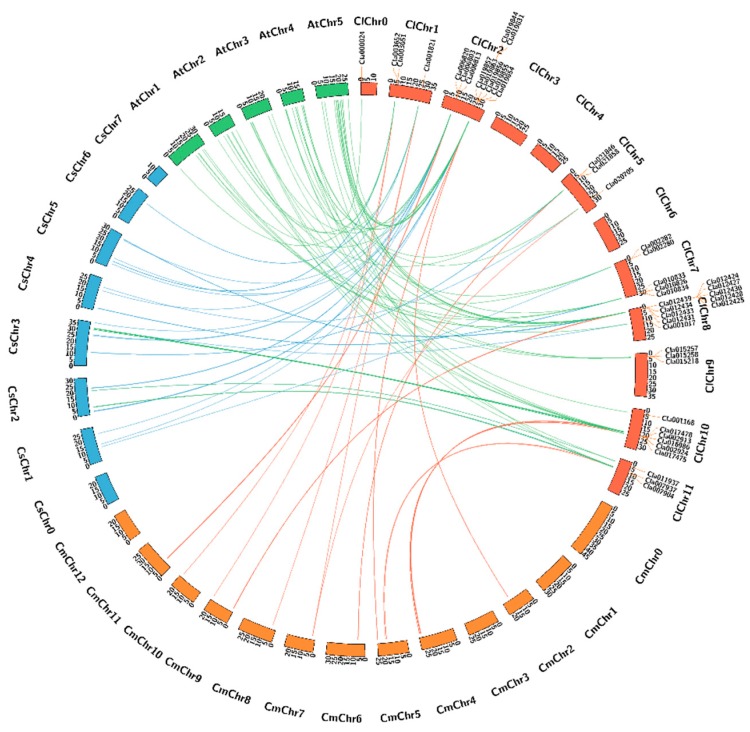
Microsynteny analysis of the 44 watermelon NBS-encoding *R* genes compared to those of *Cucumis melo*, *Cucumis sativus* and *A. thaliana*. Brown orange, blue and green indicate *C. lanatus*, *Cucumis melo*, *Cucumis sativus* and *A. thaliana* chromosomes, respectively.

We performed comparative analysis to identify the homologous NBS-encoding *R* genes among *C. lanatus*, *Cucumis melo*, *Cucumis sativus* and *A. thaliana*, with the results shown in Figure 5. Most *R* genes from *C. lanatus* share homologous relationships with those of *Cucumis melo*, *Cucumis sativus* and *A. thaliana*. However, five genes (*Cla000024*, *Cla019844*, *Cla002280*, *Cla002282* and *Cla011937*) lack homologues in *Cucumis melo* and three (*Cla000024*, *Cla019844* and *Cla002280*) lack homologues in *Cucumis sativus*. On the other hand, all 44 *R* genes of *C. lanatus* have homologues in *A. thaliana*. 

### 2.5. Expression Patterns of the NBS-Encoding R Genes in Resistant and Susceptible Watermelon Lines

**Figure 6:**
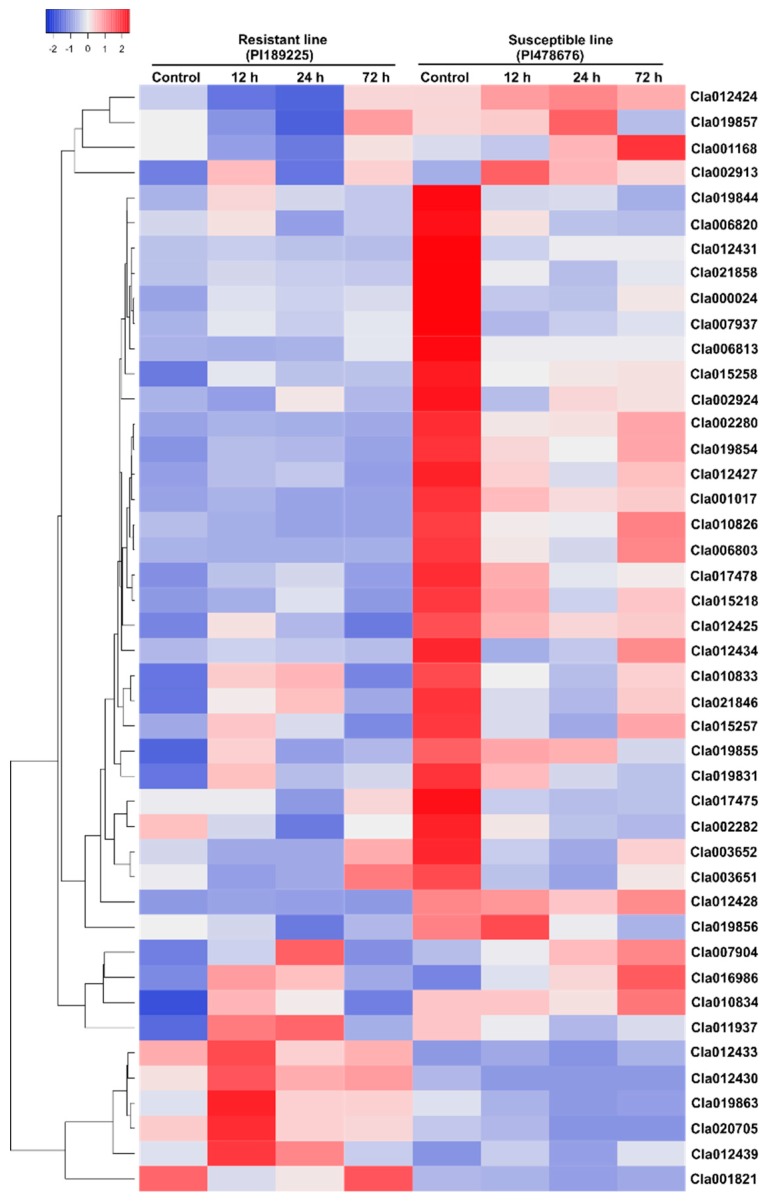
Heat map of the expression patterns of the 44 NBS-encoding *R* genes determined by qRT-PCR in gummy stem blight resistant and susceptible watermelon lines subjected to *Didymella bryoniae* infection at various time points. The expression levels were normalized with the *Actin* gene. The values were obtained from the means of three technical replicates. Red and blue represent the maximum and minimum values, respectively. The heat map was generated with an online tool ‘Heatmapper’ (http://www.heatmapper.ca/expression/).

**Figure 7:**
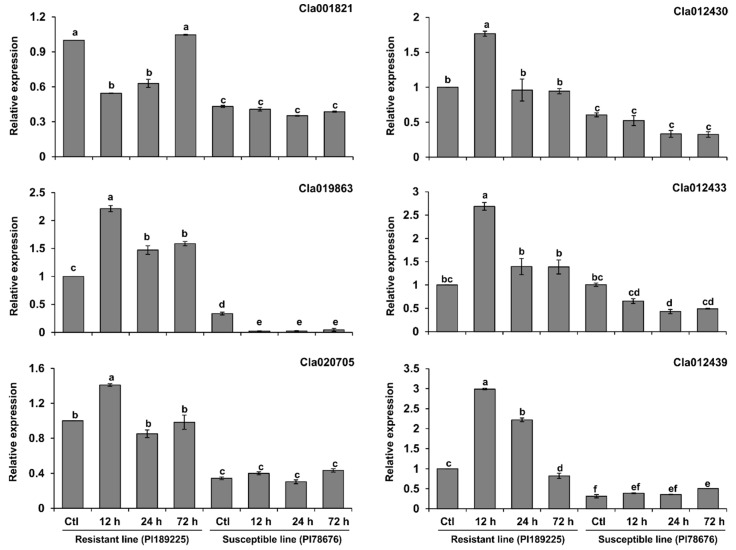
Relative expression levels of candidate NBS-encoding *R* genes in *Didymella bryoniae*-resistant and susceptible watermelon lines. Error bars represent ± SE of the means of three technical replicates. Different letters above the bars indicate significant differences.

GSB, which is one of the most devastating diseases of cucurbits, significantly reduces the yield and quality of watermelon. To gain insight into the roles of NBS-encoding genes in the response to GSB in watermelon, we designed specific primers for the 44 NBS-encoding *R* genes and analyzed their expression patterns following inoculation with *D. bryoniae* at various time points. Several genes were differentially expressed in the leaf tissue of the resistant compared to susceptible watermelon lines (Figure 6). Among these, six genes (*Cla001821*, *Cla019863*, *Cla020705*, *Cla012430*, *Cla012433* and *Cla012439*) were expressed at higher levels in the resistant line compared to the susceptible line. These genes belong to the same cluster in the heat map (Figure 6 and Figure 7). The transcript levels of five of these six genes reached a peak at 12 h postinoculation with *D. bryoniae* in the resistant line, whereas *Cla001821* transcript levels reached a peak at 72 h postinoculation. Finally, the transcript levels of *Cla020705* peaked at both 12 and 72 h postinoculation in the resistant line.

## 3. Discussion

Watermelon is an economically important fruit crop that is widely cultivated throughout the tropical and subtropical regions of the world. Watermelon is frequently affected by fungal, bacterial, viral and insect pests. GSB is a severe disease of watermelon caused by the fungus *D. bryoniae*, which significantly reduces fruit yields and quality. To reduce crop losses due to GSB, it is important to investigate the mechanism underlying the resistance to this disease. The ultimate target of both breeders and researchers is to develop GSB-resistant cultivars, since chemical treatment is not an environmentally suitable approach for controlling GSB [29]. Gene pyramiding is an effective way to increase the chances of conferring stable resistance to plant diseases, but disease resistance can break down due to increasing mutation rates in the pathogen population [9,30]. 

NBS-encoding *R* genes play important roles in plant protection against a diverse range of pathogens, including fungi, bacteria, viruses, aphids and nematodes [15,31]. For example, the *R* genes *Fom1*, *Prv* and *Vat* are responsible for resistance to *Fusarium*, Papaya ringspot virus and aphid resistance in melon, respectively [32,33]. The *RPS6* gene plays a role in resistance against the bacterium *Pseudomonas syringae* in *A. thaliana* [14]. In addition, the *R* gene Bo1037156 (*FOC1*) confers resistance to the fungal pathogen *Fusarium* in *Brassica oleracea* [34]. NBS-LRR disease resistance genes have been extensively studied in various plant species, such as *Cucumis melo* [35], *Arabidopsis* [36], *Oryza sativa* [17], *Zea mays* [37], *Solanum tuberosum* [38] and *Glycine max* [39]. However, the roles of NBS-LRR genes in response to *D. bryoniae* infection in watermelon have not been reported. The analysis of 44 NBS-encoding genes performed in this study revealed that these genes encode TIR-LRR, CC-NBS and CC-NBS-LRR proteins (Table 3). These genes are distributed throughout all watermelon chromosomes except Chr3, Chr4 and Chr6, with at least one gene per chromosome (Figure 1).

The analysis of qRT-PCR expression revealed that various genes were differentially expressed in resistant compared to susceptible watermelon. Consistent expression patterns were detected for six genes, with higher levels of expression in the resistant line compared to the susceptible line (Figure 6 and Figure 7). These candidate genes belong to different categories, including NBS-LRR (*Cla012433*), RPW8-NBS-LRR (*Cla001821*), RPW8-LRR (*Cla020705*), TIR (*Cla012430*) and TIR-LRR (*Cla012439* and *Cla019863*) (Table 3). NBS-encoding genes contribute to disease resistance in various plant species. For example, TIR-NBS-LRR-type *R* genes confer resistance to tobacco mosaic virus in *Nicotiana benthamiana* and GSB in *Cucumis melo* [5,40]. In *indica* rice, the *R* gene *Os11g0704100* (*Pia*), which encodes an NBS-LRR domain-containing protein, functions in rice blast resistance, with significantly higher expression detected in resistant compared to susceptible land races both before and after pathogen inoculation [41]. In *Arabidopsis*, the expression levels of four TIR-NBS (TN) genes increased upon treatment with different pathogens [42]. Finally, three NBS-encoding genes (*Bo1010559*, *Bo129866* and *Bo1042121*) were recently shown to function in resistance to black rot in cabbage (*Brassica oleracea* var. *capitata*), with higher expression levels detected in the resistant line compared to the susceptible lines [43].

In this study, we identified six candidate genes for GSB resistance, including *Cla012430*, *Cla012433* and *Cla012439* on Chr8 and *Cla001821*, *Cla019863* and *Cla020705* on Chr1, Chr2 and Chr5, respectively. The existence of homologues of these candidate genes in melon, cucumber and *Arabidopsis* (Figure 5) indicates that these genes likely play similar roles in these plants. Although GSB is a major yield-limiting factor for watermelon, few genetic studies have focused on this disease. Moreover, to date, no quantitative trait loci associated with GSB resistance in watermelon have been identified. To our knowledge, this is the first report of candidate genes for GSB resistance in watermelon. 

## 4. Materials and Methods

### 4.1. Experimental Materials

**Figure 8:**
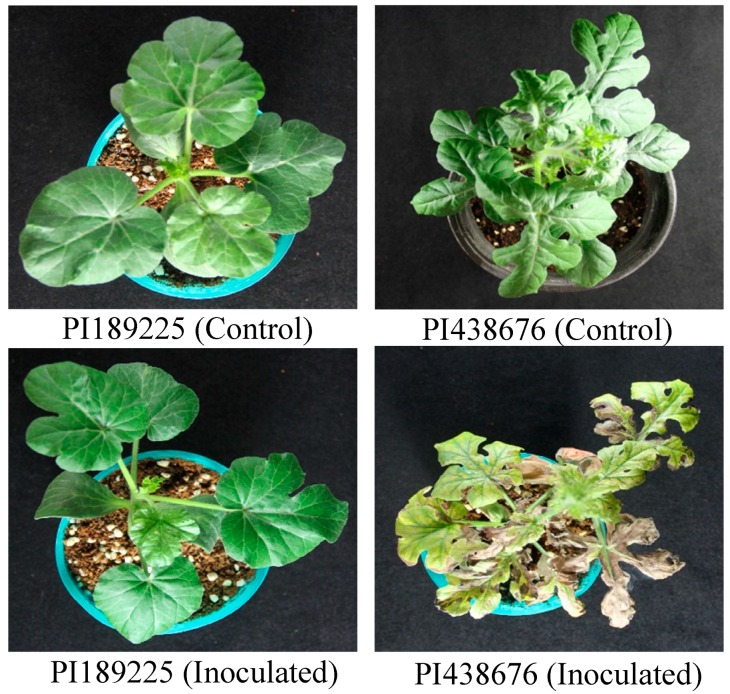
Phenotypes of watermelon lines PI189225 (resistant) and PI438676 (susceptible) after inoculation with *Didymella bryoniae*.

The GSB-resistant ‘PI189225’ and -susceptible ‘PI438676’ (Charleston Gray) watermelon inbred lines used in this study were obtained from National Plant Germplasm System (NPGS), U.S. Department of Agriculture (USDA). The resistance and susceptibility of these lines were previously assessed via bioassay screening using the fungus *D. bryoniae* [4,44]. In this study, these lines were reexamined using an intensive bioassay, which confirmed the GSB resistance of ‘PI189225’ and the susceptibility of ‘PI438676’ (Figure 8). The seeds were sown in a commercial soil mixture in a 32-hole plastic tray and transferred to a plant growth chamber at a constant temperature of 25 ± 1 °C, relative humidity of 60% and light intensity of 80–120 µmol.m^−2^·s^−1^. Two weeks after germination, the plants were transferred to plastic pots and grown in a glasshouse where plants were inoculated with *D. bryoniae*, which was maintained at 24 ± 2 °C temperature with 90% relative humidity. 

### 4.2. Pathogen Inoculation and Sampling

A *D. bryoniae* fungal isolate (13-020) was collected from National Institute of Horticulture and Herbal Sciences (NIHHS), Republic of Korea. The fungus was cultured in potato dextrose agar (PDA) medium at 24 ± 2 °C under alternating periods of 12-h fluorescent light (40–90 µmol·m^−2^.sec^−1^ PPFD) and 12-h darkness for 2–3 weeks until pycnidia formed. The final concentration of the fungal spores was adjusted to 5 × 10^5^ spores mL^−1^ with deionized water. For fungal inoculation, 28-day-old resistant and susceptible plants were inoculated with *D. bryoniae* by hand using a spray bottle, whereas control resistant and susceptible plants were sprayed with plain water. The inoculated plants were incubated in a growth chamber with a relative humidity of 90–95% and a temperature of 24 °C. Samples were collected from the third and fourth true leaves of the plants at the time points 12, 24 and 72 h after inoculation and from control plants at the same time points. The samples were immediately frozen in liquid nitrogen and stored at −80 °C.

### 4.3. Total RNA Isolation and cDNA Synthesis

Infected and control watermelon leaves were ground to a powder in liquid nitrogen and 100 mg of each sample was subjected to total RNA extraction using an RNeasy Mini kit (Qiagen, Valencia, CA) following the manufacturer’s instructions. First-strand cDNA was synthesized from total RNA with a SuperScript III First-Strand Synthesis System kit (Invitrogen, Gaithersburg, MD).

### 4.4. Exploring NBS-Encoding Genes in C. lanatus

Few genetic studies of GSB resistance in watermelon have been performed. However, 44 NBS-LRR genes have been identified by genomic analysis [1] and these genes were subjected to expression analysis in this study (Table 5).

### 4.5. Quantitative RT-PCR Analysis 

The expression patterns of the 44 NBS-encoding genes were analyzed by quantitative RT-PCR (qRT-PCR) in a LightCycler^®^ instrument (Roche, Mannheim, Germany) following the manufacturer’s instructions. The gene sequences used in this study were retrieved from the Cucurbit Genomics Database (http://cucurbitgenomics.org/) considering ‘97103’ as the reference genome for watermelon. Gene-specific primers for qRT-PCR were designed using Primer3Plus (https://primer3plus.com/cgi-bin/dev/primer3plus.cgi) (Table 5 and Table S1). The reactions were performed in a 10-µL volume consisting of 5 µL of 2× qPCRBIO SyGreen Mix Lo-ROX (PCR Biosystems, London, UK), 5 pmol of primers and cDNA templates diluted to the appropriate concentrations. The PCR conditions were as follows: 5 min at 95 °C, followed by 3-step amplifications at 95 °C for 15 s, 58 °C for 15 s and 72 °C for 20 s for 45 cycles. The relative expression level of each gene was evaluated using the comparative 2^−ΔΔ*C*T^ method, with *Actin* used as an internal control [45]. 

### 4.6. Statistical Analysis

Analysis of variance (ANOVA) and significance tests were carried out using the normalized gene expression values with MINITAB17 software (Minitab Inc., State College, PA, USA). The means were separated by Tukey’s pairwise comparisons. 

## 5. Conclusions

We identified six candidate genes that might be involved in the response of watermelon to *D. bryoniae* infection based on their expression profiles in resistant and susceptible watermelon lines. This study provides the basis for further functional studies to confirm the association of NBS-LRR genes with GSB resistance in watermelon. In addition, our results should facilitate marker-assisted breeding for developing GSB-resistant watermelon cultivars through gene pyramiding. 

## Figures and Tables

**Table 1:** Location of 44 NBS-encoding genes on the watermelon chromosomes.

Sl. No.	Gene Name ^a^	Chr	Start	End	CDS (bp)	Protein (aa)
1	*Cla000024*	Chr0	573,701	575,732	1725	574
2	*Cla001821*	Chr1	26,529,719	26,532,585	2448	815
3	*Cla003651*	Chr1	5,737,988	5,740,048	2061	686
4	*Cla003652*	Chr1	5,741,726	5,742,592	867	288
5	*Cla006803*	Chr2	9,631,689	9,634,916	3228	1075
6	*Cla006813*	Chr2	9,860,817	9,862,469	1653	550
7	*Cla006820*	Chr2	9,989,292	9,992,234	2943	980
8	*Cla019831*	Chr2	26,750,001	26,753,327	2445	814
9	*Cla019844*	Chr2	26,582,380	26,589,679	4227	1408
10	*Cla019854*	Chr2	26,456,943	26,459,976	2667	888
11	*Cla019855*	Chr2	26,449,200	26,453,033	2826	941
12	*Cla019856*	Chr2	26,439,873	26,444,126	2832	943
13	*Cla019857*	Chr2	26,432,098	26,437,657	3036	1011
14	*Cla019863*	Chr2	26,383,499	26,388,744	3588	1195
15	*Cla020705*	Chr5	27,501,959	27,505,144	2466	821
16	*Cla021846*	Chr5	6,824,170	6,829,020	1239	412
17	*Cla021858*	Chr5	6,953,920	6,957,366	3447	1148
18	*Cla002280*	Chr7	1,370,278	1,374,401	3930	1309
19	*Cla002282*	Chr7	1,381,169	1,385,954	2979	992
20	*Cla010826*	Chr7	30,211,275	30,212,050	606	201
21	*Cla010833*	Chr7	30,251,502	30,254,738	3237	1078
22	*Cla010834*	Chr7	30,256,839	30,260,099	3261	1086
23	*Cla001017*	Chr8	11,759,681	11,762,959	3279	1092
24	*Cla012424*	Chr8	1,647,030	1,648,988	1377	458
25	*Cla012425*	Chr8	1,641,639	1,643,644	1299	432
26	*Cla012427*	Chr8	1,609,802	1,613,337	3042	1013
27	*Cla012428*	Chr8	1,595,322	1,601,397	4353	1450
28	*Cla012430*	Chr8	1,580,252	1,584,048	1278	425
29	*Cla012431*	Chr8	1,547,018	1,555,932	4578	1525
30	*Cla012433*	Chr8	1,530,729	1,534,838	2454	817
31	*Cla012434*	Chr8	1,420,444	1,427,949	3423	1140
32	*Cla012439*	Chr8	1,331,566	1,335,184	2469	822
33	*Cla015218*	Chr9	3,429,200	3,430,883	1404	467
34	*Cla015257*	Chr9	3,116,792	3,119,902	3111	1036
35	*Cla015258*	Chr9	3,111,030	3,115,364	3444	3444
36	*Cla001168*	Chr10	4,050,530	4,052,711	1050	349
37	*Cla002913*	Chr10	21,882,174	21,883,565	1392	463
38	*Cla002924*	Chr10	21,745,526	21,748,738	3213	1070
39	*Cla016986*	Chr10	21,332,030	21,336,749	3402	1133
40	*Cla017475*	Chr10	22,939,927	22,942,518	2592	863
41	*Cla017478*	Chr10	22,955,431	22,957,062	1632	543
42	*Cla007904*	Chr11	9,507,150	9,514,341	4062	1353
43	*Cla007937*	Chr11	8,764,524	8,779,874	3391	1155
44	*Cla011937*	Chr11	3,833,117	3,836,951	1725	574

^a^ Genomic information retrieved from the Cucurbit Genomics Database (http://cucurbitgenomics.org) using 97103 as the reference genome for watermelon.

**Table 2:** Key domains in the 44 watermelon NBS proteins.

Sl. No.	Domain Name	Description	Function	Reference
1	RPW8	Resistance to Powdery Mildew8	Involved in powdery mildew resistance	[23]
2	LRR	Leucine-rich repeats	Disease resistance	[24]
3	NB-ARC	Nucleotide-binding adaptor shared by APAF-1, R proteins and CED-4	Disease resistance	[25,26]
4	TIR	Toll-interleukin 1-receptor	Plant defense	[25]
5	CC	Coiled-coiled	Disease resistance	[27,28]

**Table 3:** Classification of the 44 *Citrullus lanatus* NBS-encoding genes.

Sl. No.	Type	Gene Name
1	NBS	*Cla003652*, *Cla002280*, *Cla002282* and *Cla001168*
2	NBS-LRR	*Cla001821*, *Cla003651*, *Cla006820*, *Cla019831*, *Cla012433* and *Cla017478*
3	LRR	*Cla002924*, *Cla011937* and *Cla000024*
4	RPW8-NBS-LRR	*Cla001821*, *Cla019831* and *Cla020705*
5	TIR	*Cla012424*, *Cla012425* and *Cla012430*
6	TIR-LRR	*Cla019854*, *Cla019855*, *Cla019856*, *Cla019857*, *Cla019863*, *Cla012427*, *Cla012428*, *Cla012431*, *Cla012439*, *Cla016986* and *Cla007937*
7	CC-NBS	*Cla006813*, *Cla010826*, *Cla015218* and *Cla002913*
8	CC-NBS-LRR	*Cla006803*, *Cla021858*, *Cla010833*, *Cla010834*, *Cla001017*, *Cla015257* and *Cla017475*

**Table 4:** Putative conserved motifs in the 44 watermelon NBS proteins.

Motif Name	E-Value	Sites	Width	Motif Sequence
Motif 1	1.2 × 10^−386^	38	29	DDVRFVGIVGMGGIGKTTLAKAVYNHILI
Motif 2	2.30 × 10^−290^	40	29	LLDLSKEIVKYCGGLPLAJKVLGSSLRGK
Motif 3	1.70 × 10^−232^	17	37	IIKNRLSSKKVLJVLDDVDELEQLZALAGGRDWFGPG
Motif 4	1.50 × 10^−220^	33	29	JKELPESIGNLTSLKTLNLKNCSNLKELP
Motif 5	1.30 × 10^−204^	15	29	YDVFLSFRGEDTRRNFTSHLYEALRQKGI
Motif 6	4.90 × 10^−189^	15	29	VLPVFYKVDPSDVRKQTGSFGZAFAKHEA
Motif 7	1.70 × 10^−187^	34	21	KVKMHDLIQDMARTIVRKZSV
Motif 8	1.80 × 10^−167^	37	21	YNVEKLSDEEALELFSKHAFG
Motif 9	3.40 × 10^−119^	35	13	SKIIVTTRNEHLL
Motif 10	1.00 × 10^−117^	21	21	PSSSLKQCFLYCSLFPKDYKF
Motif 11	1.10 × 10^−171^	28	41	PDFSSLPNLEKLDJEGCTNLVKLHESIGSLKKLIKLDJKDC
Motif 12	1.50 × 10^−157^	8	50	MAEFLWTFAVZEVLKKTLKLAAEQIGLAWGFKKELSKLRKSLLKVEAILR
Motif 13	7.60 × 10^−122^	13	21	FSENYASSKWCLEELVKIIEC
Motif 14	2.10 × 10^−126^	22	29	KHFDKVIWVCVSZPFDVKKILEEIJESLN
Motif 15	1.10 × 10^−109^	15	27	KEIFLDIACFFKGEDVELVKEILEACG
Motif 16	6.60 × 10^−92^	11	29	EHHSVKJWVEKLZDIVYEADDLLDELSYE
Motif 17	8.20 × 10^−90^	10	34	NSSGGLDSKEALLRELQKELHGKRYFLVLDDVWN
Motif 18	5.70 × 10^−92^	11	29	TMEDIGDKYFNELLSRSLFQDVVKDKRGR
Motif 19	4.90 × 10^−78^	13	29	SWHGFPFKSLPSDFHPENLVELDLRYSCI
Motif 20	7.60 × 10^−97^	22	29	SLRVLDLSNTNITKLPNSIGQLKHLRYLD

**Table 5:** Primers for the 44 NBS-encoding genes in watermelon.

Sl. No.	Gene Name	Forward Primer	Reverse Primer	Product Size (bp)
1	*Cla001821*	ACTGTCTAACGAGTCTTGAACG	TTCTCCAGATTTATCAGCGGT	94
2	*Cla003651*	ATCTCTCGATTATGTGGGCGG	TTGGGTGGCACGGTTACTCTG	118
3	*Cla003652*	TCCCAAAATCGCTCTTCTGC	AGATACCGTTTGCCTCTCAGT	126
4	*Cla006803*	GGAAACGATCCAAACCATAGG	CTTCCCTTTGCTCCAAGTTGA	85
5	*Cla006813*	AACTCATGGAAACACGCCCTA	CCAATGGTACGCCACCAACT	165
6	*Cla006820*	AGGTTGTATCAGAGATGAGTTC	CATATTCATTAGAAGCCGTGGGT	108
7	*Cla019831*	GGAGTGTTTCATGGACTTGG	TTCATGTGCTTCGTTTCTCA	178
8	*Cla019844*	AGTGTTGAAGGAAGAGCAGCC	TAAATCAAGTCCCTCCCACA	96
9	*Cla019854*	ACAAATGGGTCGCACAATCGC	TGGCTTTAACTGCTCTTGCT	131
10	*Cla019855*	GTGAGGTACGAAAACAAACTGG	ACAACCCAGCAGCAGTAGTC	122
11	*Cla019856*	GTCAACAAACATCCGAGGATTA	TCAAGGTTTAATGTCGCCGAG	116
12	*Cla019857*	CCAGTCTTCCTTGTTGGATGG	CCATCCAACAAGGAAGACTGG	171
13	*Cla019863*	GTATAGGAAGCTGTGGCGTCC	TCCCCAGTGTCCGGATTTTGC	127
14	*Cla020705*	GTTGTCACGAGGCGAGCCTAT	TTTGTCTTCAGGAAAGCATCCC	133
15	*Cla021846*	CCTGACCCATCTTCTCCTATT	ATCTTGCACTTTCGCATGAACA	135
16	*Cla021858*	GAAACCATGGAAGACATAGGAG	TGAGACAGAATAAGCAAGATCG	144
17	*Cla002280*	AGATGGGGAGCGGAAGTATCA	CCCATTTCTATACTCAACTCAG	146
18	*Cla002282*	GGTTAGAAGATGATAGACGTGG	GGAAACTCCAACTCCAGGGGA	91
19	*Cla010826*	TGTGGCATGGGGCTTGGACA	GATCCTTCACCCACAATCTCAC	128
20	*Cla010833*	AGTGTGGATCGCCTACCGTCA	CATTCCTTTCCTCAGATGGTCG	145
21	*Cla010834*	CTCCGACCCTTGTGATGCTA	ACATCCATCATCAAACCCAACT	100
22	*Cla001017*	GCAAACATTGTGGAGACGCAT	CAAGCACTTCTCGAACTACA	145
23	*Cla012424*	GTGGGGATGTGTAGTATTGGTA	ATCAATGCGAAGGAAGCAACTA	99
24	*Cla012425*	AGCGGGAGGTGATTCAAAGC	GAACCTTTCTACCACTCAGTCG	143
25	*Cla012427*	CAGCCGAGTGAACTATTGGAAC	GCACCATTGACAGATCAGGG	145
26	*Cla012428*	TCCAGCATTCGATGTTTACTCG	ACCATTCCAATAGGTCAACATC	99
27	*Cla012430*	TGGTTGGAATTAGCCACAGATT	CCAATTCCACCCATTCCCCATA	100
28	*Cla012431*	GCTTGGATCCTCAATCACAGTC	GAGAGTTTTGTAGCACTTGGAAG	116
29	*Cla012433*	GGATTTGGATGAGGAAGGAGAA	GTATCCATGCCAATTGAGAAAC	151
30	*Cla012434*	CTCTCATCTTCCAACAAGCATA	TCCTTGGAGGTAGCTCTGGGA	101
31	*Cla012439*	GCGGCATTGGCAAGACGACA	AATAAGGAAGATTGTGGGTCTC	120
32	*Cla015218*	GTTGAAAGGGTCTCCTCTTGC	GTGCAAAGTTCACTGTCCTTGA	102
33	*Cla015257*	GGGAAGTTGAGTTGTCTACAGA	CTAAACTGACTTCAGATGGACT	157
34	*Cla015258*	GTGGAGTCAAATTTCCCAACTG	CCAAATATAGAGAATGGAGAGC	132
35	*Cla001168*	GTGTACCAAGCGTTTGGAGTT	TACTTAACCTGCCCCTCAACT	163
36	*Cla002913*	AGAATGGCTTATGGGACGAGC	TGGTTGCCACTTCTAGGTTCC	102
37	*Cla002924*	CTCAATTCCCTTCAAACACTGA	GCTCACACCCCCATATTGCC	112
38	*Cla017475*	GGTCGTTACCGGAAGATACTCA	CATCTAGCTCATTCAGAGGGC	123
39	*Cla017478*	CCCAGTCACAGAACCTAAATCT	CAGCATTTGGTATTTCCCGTAG	149
40	*Cla016986*	CTTTTGGAAGGAGTGTAAGTTG	TTCTCTAGATTTGGGAGTTTGG	108
41	*Cla007904*	GCAGCATCCACACAGTGCCTT	GGAAAGACATTTGTGGAAGCC	104
42	*Cla007937*	AAGATCGACCGCCTCCACGA	CCGATCTTCTGCAGTTACAAC	154
43	*Cla011937*	GAGAGGATGTTGAACTTGCCAG	GCTTTCCACATCTCTTGAATCA	154
44	*Cla000024*	GACATTGAAGGCATAGTGATGG	CCATGCCAATTGAGAAACCTC	167
Actin	*Cla007792*	CCATGTATGTTGCCATCCAG	GGATAGCATGGGGTAGAGCA	140

## Supplementary Materials

Supplementary materials can be found at http://www.mdpi.com/1422-0067/20/4/902/s1.
