# Supplementary material for: Genome-Wide Characterization of NBS-Encoding Genes in Watermelon and Their Potential Association with Gummy Stem Blight Resistance
Source: Int J Mol Sci. 2019 Feb 19;20(4):902. doi: 10.3390/ijms20040902 (PMC6412240; doi:10.3390/ijms20040902)
Supplement: Supplementary file 1 [file ijms-20-00902-s001.pdf]

Table S1. Primer specification and efficiency for the 44 watermelon NBS-encoding genes.

Sl. no.	Gene ID	Tm (Forward primer)	Tm (Reverse primer)	Efficiency (%)
1	Cla001821	53	50	91.84
2	Cla003651	54	56	91.33
3	Cla003652	52	52	99.88
4	Cla006803	52	52	99.41
5	Cla006813	52	54	90.91
6	Cla006820	51	53	99.90
7	Cla019831	52	48	105.82
8	Cla019844	54	50	101.77
9	Cla019854	54	50	96.41
10	Cla019855	53	54	94.90
11	Cla019856	51	52	107.76
12	Cla019857	54	54	100.20
13	Cla019863	56	57	101.10
14	Cla020705	56	53	100.21
15	Cla021846	52	51	100.00
16	Cla021858	53	51	100.95
17	Cla002280	54	51	99.80
18	Cla002282	53	56	98.08
19	Cla010826	56	55	97.18
20	Cla010833	56	55	98.90
21	Cla010834	54	51	92.20
22	Cla001017	52	50	99.20
23	Cla012424	53	51	103.4
24	Cla012425	54	55	90.63
25	Cla012427	55	54	110.77
26	Cla012428	53	51	99.88
27	Cla012430	51	55	108.73
28	Cla012431	55	53	90.91
29	Cla012433	53	51	99.86
30	Cla012434	51	56	95.72
31	Cla012439	56	51	101.77
32	Cla015218	54	53	94.80
33	Cla015257	53	51	99.29
34	Cla015258	53	51	98.52
35	Cla001168	52	52	91.09
36	Cla002913	54	54	98.88
37	Cla002924	51	56	105.99
38	Cla017475	55	54	100.66
39	Cla017478	53	53	98.88
40	Cla016986	51	51	94.99
41	Cla007904	56	52	93.26
42	Cla007937	56	52	100.19
43	Cla011937	55	51	98.90
44	Cla000024	51	53	100.63
Actin	Cla007792	52	54	99.17
